# Efficacy of Dabrafenib and Trametinib in a Patient with Squamous-Cell Carcinoma, with Mutation p.D594G in *BRAF* and p.R461* in *NF1* Genes—A Case Report with Literature Review

**DOI:** 10.3390/ijms24021195

**Published:** 2023-01-07

**Authors:** Anna Grenda, Pawel Krawczyk, Katarzyna M. Targowska-Duda, Robert Kieszko, Iwona Paśnik, Janusz Milanowski

**Affiliations:** 1 Department of Pneumonology, Oncology and Allergology, Medical University in Lublin, Jaczewskiego 8, 20-090 Lublin, Poland; 2 Department of Biopharmacy, Medical University in Lublin, Chodźki 4a, 20-093 Lublin, Poland; 3 Department of Clinical Pathomorphology, Medical University in Lublin, Jaczewskiego 8b, 20-090 Lublin, Poland

**Keywords:** squamous-cell carcinoma, *BRAF* mutation, *NF1* mutation, dabrafenib, trametinib, *FANCC* mutation

## Abstract

The 3rd class of *BRAF* (B-Raf Proto-Oncogene, Serine/Threonine Kinase) variants including G466, D594, and A581 mutations cause kinase death or impaired kinase activity. It is unlikely that RAF (Raf Proto-Oncogene, Serine/Threonine Kinase) inhibitors suppress ERK (Extracellular Signal-Regulated Kinase) signaling in class 3 mutant-driven tumors due to the fact that they preferentially inhibit activated BRAF V600 mutants. However, there are suggestions that class 3 mutations are still associated with enhanced RAS/MAPK (RAS Proto-Oncogene, GTPase/Mitogen-Activated Protein Kinase) activation, potentially due to other mechanisms such as the activation of growth factor signaling or concurrent MAPK pathway mutations, e.g., RAS or NF1 (Neurofibromin 1). A 75-year-old male patient with squamous-cell cancer (SqCC) of the lung and with metastases to the kidney and mediastinal lymph nodes received chemoimmunotherapy (expression of Programmed Cell Death 1 Ligand 1 (PD-L1) on 2% of tumor cells). The chemotherapy was limited due to the accompanying myelodysplastic syndrome (MDS), and pembrolizumab monotherapy was continued for up to seven cycles. At the time of progression, next-generation sequencing was performed and a c.1781A>G (p.Asp594Gly) mutation in the *BRAF* gene, a c.1381C>T (p.Arg461Ter) mutation in the *NF1* gene, and a c.37C>T (p.Gln13Ter) mutation in the *FANCC* gene were identified. Combined therapy with BRAF (dabrafenib) and MEK (trametinib) inhibitors was used, which resulted in the achievement of partial remission of the primary lesion and lung nodules and the stabilization of metastatic lesions in the kidney and bones. The therapy was discontinued after five months due to myelosuppression associated with MDS. The molecular background was decisive for the patient’s fate. NSCLC patients with non-V600 mutations in the *BRAF* gene rarely respond to anti-BRAF and anti-MEK therapy. The achieved effectiveness of the treatment could be related to a mutation in the *NF1* tumor suppressor gene. The loss of NF1 function causes the excessive activation of KRAS and overactivity of the signaling pathway containing BRAF and MEK, which were the targets of the therapy. Moreover, the mutation in the *FANCC* gene was probably related to MDS development. The NGS technique was crucial for the qualification to treatment and the prediction of the NSCLC course in our patient. The mutations in two genes—the *BRAF* oncogene and the *NF1* tumor suppressor gene—were the reason for the use of dabrafenib and trametinib treatment. The patients achieved short-term disease stabilization. This proved that coexisting mutations in these genes affect the disease course and treatment efficacy.

## 1. Introduction

### BRAF Mutation Characteristics

Changing the nucleotide A to G in the 1781 nt position of the *BRAF* gene results in the replacement of aspartic acid by glycine (p.Asp594Gly, p.D594G) in the BRAF protein chain. It is described as a pathogenic variant or a variant of unknown significance (VUS) in COSMIC (*Catalogue of Somatic Mutations in Cancer*) and ClinVar databases. This variant has been reported as a somatic molecular change in large intestine, skin, and stomach cancers and has been observed in up to 11% of *BRAF* mutations in lung adenocarcinoma [1,2]. The genetic variant identified in our patient is localized close to the most common drug response type mutation p.V600E (p.Val600Glu). Mutations other than changes in the 600th codon are found in 1–3% of all cancers, including many common histological types (lung, colon, prostate, gynecologic malignancies) as well as less common tumor types (primary brain tumors, neuroendocrine tumors, and hematologic malignancies) [3]. Zheng et al. [4] examined *BRAF* mutations in lung cancer, colorectal cancer, and melanoma specimens and observed that mutations other than changes in the 600th codon occurred with the frequency of 37% of all mutations in the *BRAF* gene [4]; 75% of all cases with *BRAF* mutations were kinase-activating variants, 15% were kinase-impairing variants, and 10% had unknown kinase effects [4]. Lung tumors showed a much higher incidence of kinase-impaired mutants or mutants with an unknown impact on the *BRAF* gene. *BRAF* variants with kinase impairment showed a significant association with co-occurring activating mutations of the *KRAS* or *NRAS* genes [4].

*BRAF* mutations are categorized into three classes: the 1st type of mutations, which are exclusively *BRAF* V600 mutations, the 2nd type of mutations including L597 and K601 (intermediate or high kinase activity) and, finally, the 3rd class of *BRAF* variants including G466, D594, and A581 mutations causing kinase death or impaired kinase activity [3,5]. It is unlikely that RAF inhibitors suppress ERK signaling in class 3 mutant-driven tumors due to the fact that they preferentially inhibit activated BRAF V600 mutants. There are suggestions that class 3 mutations are still associated with enhanced RAS/MAPK activation, potentially due to other mechanisms such as the activation of growth factor signaling or concurrent MAPK pathway mutations, e.g., *RAS* or *NF1* [3,5]. Here, we present a case with an identified lesion other than V600E in *BRAF* gene, in which treatment with trametinib and dabrafenib was effective.

## 2. Case Description

**Scheme 1:**
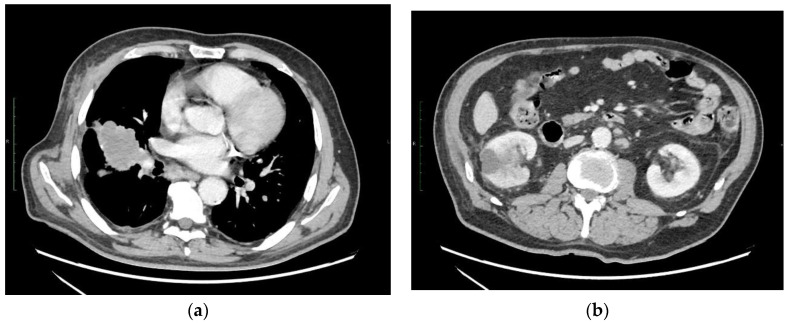
Computed tomography showing the tumors of the apex of the right lung with metastases to mediastinal and hilar lymph nodes (**a**) as well as a metastatic lesion in the right kidney (**b**).

Tumors of the apex of the right lung and the right kidney with metastases to the mediastinal and hilar lymph nodes and bones were simultaneously diagnosed in a smoking male patient at the age of 75 years (Scheme 1a,b). Renal biopsy specimens revealed poorly differentiated squamous-cell carcinoma (immunohistochemical analysis described later in the manuscript). The material from the subsequent thoracotomy also revealed squamous-cell carcinoma (p40 (+), CK7 (+), TTF1 (−), CK20 (−), CD10 (−), Vimentin (−), Napisn A (−), PAX8 (−)). PD-L1 (programmed death ligand 1) expression was found in 2% of tumor cells. The patient’s history of comorbidities included post myocardial infarction status, type 2 diabetes mellitus, myelodysplastic syndrome (MDS), and gout.

After the oncological consultation, the patient received chemoimmunotherapy with a regimen of carboplatin, paclitaxel, and pembrolizumab.

Patients with advanced squamous lung cancer who remain in good performance status should be treated in the first line with chemoimmunotherapy or immunotherapy. Chemoimmunotherapy regimens include pembrolizumab in combination with carboplatin and paclitaxel or nab-paclitaxel, and nivolumab plus ipilimumab in combination with two cycles of platinum-based chemotherapy. Pembrolizumab or cemiplimab can be used in patients with SqCC with PD-L1 expression in ≥50% of cancer cells, and atezolizumab in patients with PD-L1 expression in ≥50% of cancer cells or ≥10% of immune cells. Platinum-based first-line chemotherapy alone may be used only in the case of contraindications to the use of immunotherapy. The testing of *EGFR*, *ALK* and *ROS1* genes as well as other genetic predictive factors is indicated in non-smoker SqCC patients. Molecularly targeted treatment should be used in the first line of treatment when these genetic abnormalities are detected. Second-line platinum-based chemotherapy is used in patients who have received immunotherapy in monotherapy in the first line. Docetaxel in monotherapy or in combination with ramucirumab (anti-EGFR antibody) is the most widely used in second-line treatment in patients treated with first-line chemoimmunotherapy and in third-line treatment in patients receiving immunotherapy followed by platinum-based chemotherapy. Atezolizumab or nivolumab or pembrolizumab may be used in the second line of treatment if, most often for non-medical reasons, immunotherapy was not used in the first line of treatment.

**Scheme 2:**
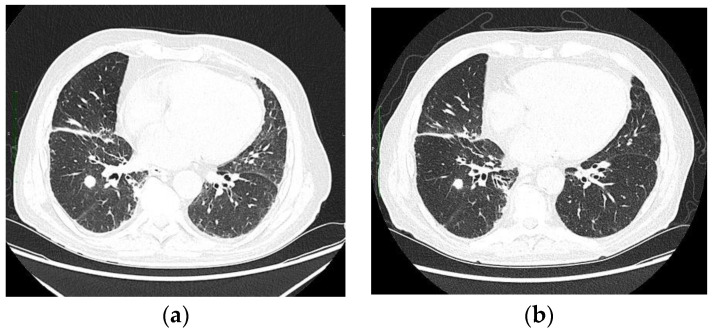
Computed tomography performed at the time of progression during pembrolizumab maintenance therapy (**a**) and CT scans showing the effectiveness of the subsequent treatment with dabrafenib and trametinib (**b**).

Due to coexisting MDS and neutropenia, the decision to introduce chemoimmunotherapy was postponed, and a granulocyte growth factor was administered. The patient received two cycles of reduced-dose chemotherapy with pembrolizumab. Unacceptable complications including thrombocytopenia developed during the chemoimmunotherapy. Due to the myelodysplastic syndrome and bone marrow dysfunction, further chemotherapy was abandoned. The patient continued the pembrolizumab therapy for seven cycles, after which the progression of the disease in the chest (an increase in the size of the primary tumor and the appearance of numerous metastatic nodules in both lungs), abdomen (enlargement of renal tumor), and the skeleton were observed (Scheme 2a). The patient also received radiotherapy for bone metastases and was treated with bisphosphonate for these metastases.

Therefore, a tumor specimen of the lung collected from the patient was subjected to next-generation sequencing with the use of the FOUNDATIONONE^®^ CDx assay, which revealed mutations with a known and unknown pathogenic status. First of all, a mutation in the *BRAF* (*B-Raf proto-oncogene, serine/threonine kinase*) gene (NM_004333) was identified in FFPE (formalin-fixed paraffin-embedded) material from the lung. There was a substitution of c.1781A>G (p.Asp594Gly, p.D594G) in exon 15 of this gene. The second clinically significant mutation was c.1381C>T (p.Arg461Ter, p.R461*) in the neurofibromin 1 (*NF1*) gene (NM_001042492). The third significant genetic change was a c.37C>T mutation in the *FANCC* (*FA Complementation Group C*) gene (p.Gln13Ter). Treatment with dabrafenib and trametinib was administered and proved to be effective with a decrease in the size of the pulmonary tumors and stabilization of bone and renal metastases (Scheme 2b). Treatment with this regimen lasted five months. Compared to the previous computed tomography performed at the time of progression during pembrolizumab maintenance therapy (Scheme 2a), the tumor mass in the hilum of the right lung (63 × 45 mm^2^) was stable (Scheme 2b). A reduction in metastatic lesions compared to the previous examination was shown: the lesion in segment 6 of the right lung from 24 mm to 21 mm, nodule in segment 1/2 of the left lung from 11 mm to 9 mm, and nodule in segment 2 of the right lung from 12 mm to 7 mm. A reduction in the dimensions of the lymph node below the main bronchus of the right lung from 26 mm to 20 mm was also observed. Disease stabilization was achieved according to RECIST 1.1 (Response Evaluation Criteria in Solid Tumors). The disease stabilization lasted for five months. After this time, the patient’s performance status deteriorated due to the reduction in the number of erythrocytes and platelets in the course of MDS. The treatment was discontinued. The disease progressed rapidly and death occurred 6.5 months after starting the therapy with BRAF and MEK inhibitors.

The patient gave his written consent to participate in the research based on the consent of the local bioethics committee at the Medical University of Lublin (No. KE-0254/160/2021).

## 3. Discussion

**Figure 1:**
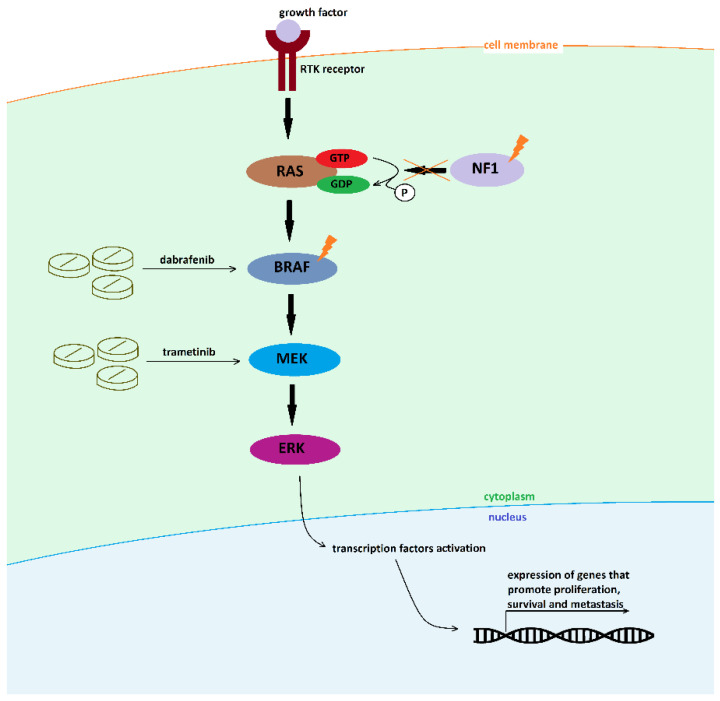
Scheme of the signal transduction pathway involving BRAF and NF1.

**Figure 2:**
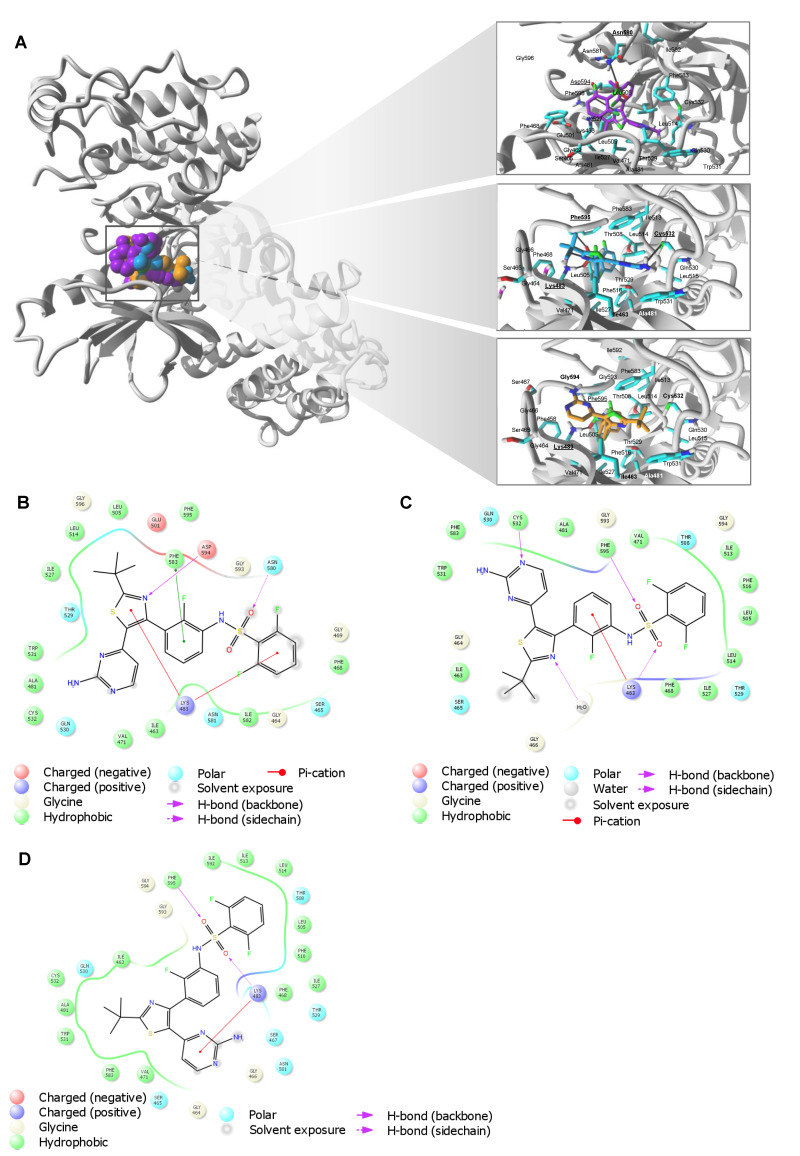
Molecular docking of dabrafenib to the human BRAF kinase V600E or D594G mutant model. (**A**) Dabrafenib binding site (purple) in the BRAF kinase V600E mutant is presented as the energetically lowest orientation. Moreover, two of the energetically lowest orientations (orange and light blue) are shown in the complex with the BRAF kinase D594G mutant model. On the right, 3D views of dabrafenib interactions with residues involved in binding. Hydrogen bonds are marked with black arrows and residues are underlined. Ligand orientations are rendered as a ball mode (on the left) and as a stick mode (on the right), whereas residues involved in ligand binding (on the right) are rendered as a stick mode (element color code with nitrogen presented in blue, oxygen in red, and hydrogens in white). (**B**) 2D views of dabrafenib interacting with the binding site in the BRAF kinase V600E mutant and (**C**,**D**) 2D views of dabrafenib binding in two (higher-affinity and lower-affinity) orientations in the BRAF kinase D594G mutant. Residues involved in dabrafenib binding are presented in panels (**B**–**D**). Hydrogen bonds are marked with pink arrows, residues involved in hydrophobic interactions.

The targeted treatment in the patient described here involved BRAF and MEK inhibitors. The efficacy of dabrafenib in combination with trametinib was proven in a phase 2 study in pretreated (cohort B) and treatment-naive (cohort C) patients with the p.V600E mutation in the *BRAF* gene. The overall response rate was 68.4% and 63.9%, the median progression-free survival was 10.2 and 10.8 months, and the median overall survival was 18.2 and 17.3 months in patients from cohorts B and C, respectively [6]. It has been shown that the treatment with a single BRAF inhibitor (vemurafenib) in lung cancer patients with non-V600E mutations is rather ineffective due to reduced or no affinity of the drugs for non-V600E mutant forms of BRAF [7]. However, there are reports that treatment with vemurafenib can lead to a partial response in melanoma patients with a p.L597R mutation in the *BRAF* gene [8]. The BRAF and MEK blockade with dabrafenib and trametinib may be effective even in patients with non-V600 mutations in the *BRAF* gene due to the effective blockade of the MAPK (MEK) pathway by trametinib (Figure 1). Negrao et al. [9] identified unique BRAF alterations, including amplifications and missense mutations in non-small-cell lung cancer patients, using the NGS technique (Guardant 360). Three patients with non-V600 mutations were treated with BRAF and MEK inhibitors. The patients with p.G469V and p.D594G mutations had rapid disease progression, while the patient with a p.L597R mutation had an ongoing partial response [9]. A study conducted by Mu et al. [10] showed that an NSCLC patient with a T599dup mutation treated with dabrafenib plus trametinib after the failure of chemotherapy with platinum and pemetrexed achieved disease stabilization (SD), while a patient with the K601E variant had disease progression (PD) [10]. The D594G mutation in exon 15 of the *BRAF* gene (like in our patient) most likely impairs but does not deactivate BRAF kinase (Figure 2). The protein can still transmit signals, most likely enhanced by RAS overactivation resulting from the deactivation of NF1, which can turn off this protein in the wild type. Thus, it appears that the synergistic effects of dabrafenib, which acts weakly on cancer cells with a non-V600 BRAF mutation, and trametinib, which blocks MEK kinase, arrest the proliferative signals and cause tumor shrinkage.

### 3.1. Molecular Docking of Dabrafenib in Human BRAF Kinase p.V600E or p.D594G Mutant Models

The human BRAF kinase in complex with dabrafenib at 2.66 Å atomic resolution (PDB ID: 5CSW) [11] was used to build the p.D594G mutant model. In addition, a crystal structure of the BRAF kinase p.V600E mutant at 2.65 Å atomic resolution (PDB ID: 4FK3) [12] was used for molecular modeling studies. The molecular structure of dabrafenib was taken from the crystallographic structure of the BRAF kinase in complex with dabrafenib as described above. Then, this molecule was geometrically optimized using the semi-empirical AM1 method found in Spartan 10V.1.1.0 (Wavefunction, Inc., Irvine, CA, USA). AutoDock Vina v.1.2.0 [13] was used for the molecular docking of the flexible dabrafenib molecule into both models of BRAF kinase carrying either a p.D594G or p.V600E mutation. The grid box size for docking was generated using MGL-AutoDockTools 1.5.6. The selected grid box dimension was 34 Å × 30 Å × 30 Å with a grid-point spacing of 1 Å to cover the dabrafenib binding site determined experimentally for human BRAF kinase [11]. The parameters used with AutoDock Vina were exhaustiveness (570) and the number of modes (20) as described previously [14]. The energetically lower poses were selected and are presented in Figure 2.

The molecular docking results indicated the location of possible binding sites and structural components for dabrafenib binding in BRAF kinase models carrying either the p.D594G or p.V600E mutation (Figure 2A). In the case of the BRAF kinase p.V600E mutant model, dabrafenib interacted with hydrophobic (Phe468, Ile463, Val471, Ala481, Leu505, Leu514, Ile527, Trp531, Cys532, Ile582, Phe583, and Phe595) and polar residues (Ser465, Thr529, Gln530, Asn581, and Asn580). In particular, cation–π interactions were suggested between Lys483 and the fluorophenyl moiety and the 1,3-thiazole moiety. Two hydrogen bonds were suggested between the ligand and Asp594 and Asn580 (Figure 2B).

In the case of the BRAF kinase p.D594G mutant model, the docking results suggested dabrafenib binding to the same site as for the V600E mutant, but several different orientations were proposed, as presented in Figure 2A. More specifically, in each presented orientation, the molecule interacted with hydrophobic (Phe468, Ile463, Val471, Ala481, Leu505, Ile513, Leu514, Phe516, Ile527, Trp531, Cys532, Ile582, Phe583, and Phe595) and polar (Ser465, Thr508, Thr529, and Gln530) residues. In each orientation, at least two hydrogen bonds were suggested between dabrafenib and Lys483 and Phe595 (Figure 2C,D). Cation–π interactions with the Lys483 residue and the fluorophenyl or 2-amino-4-pyrimidinyl moiety in each orientation in the BRAF kinase p.D594G mutant model were suggested.

To summarize, the molecular docking results were in agreement with clinical data. In particular, the same binding site was suggested for dabrafenib in both BRAF kinase p.V600E and p.D594G mutant models.

### 3.2. Molecular Background for the Patient with the BRAF Mutation

In our patient, a change in exon 12 of the *neurofibromin 1* gene (NM_001042492) c.1381C>T (p.Arg461Ter, pR461*) was identified in the FFPE tumor tissue. This mutation has a pathogenic status and is described to be associated with neurofibromatosis type 1 and hereditary familial cancer syndrome (Ref. [15], ClinVar). A nucleotide substitution of C to T results in changes in the amino acid from an arginine to a stop codon. The *NF1* gene is a canonical member of the RasGAP family [16]. Loss of function by mutation has been described in lung cancer and many other cancers [17]. The inactivation of the NF1 function results in the hyperactivation of RAS proteins by keeping them in the GTP-bound form [17]. Excessive activation of the TRK (tyrosine kinase receptor)–KRAS–BRAF–MEK–ERK pathway may explain the effectiveness of dabrafenib and trametinib in our patient. Therefore, the efficacy of BRAF and MEK inhibitors may be caused by the inactivation of NF1 and not by the mutation of *BRAF*. It cannot be ruled out that both genetic changes influenced the effectiveness of these drugs. The results of the molecular docking of dabrafenib to BRAF kinase changed by the p.V600E and p.D594G mutations presented above confirm that, in the presence of the p.D594G mutation, the binding ability of dabrafenib to BRAF kinase remains unchanged.

It has been suggested that the majority of lung cancer patients with *NF1* mutations are men and smokers, and NSCLC patients with mutated *NF1* represent a distinct molecular and clinical subtype of lung adenocarcinoma [18]. Redig et al. [19] tested 591 NSCLC patients using next-generation sequencing. Mutations in the *NF1* gene were observed in 60 (10%) patients and *KRAS* mutations were found in 141 (24%) patients. Seventy-two genetic variants of *NF1* have been identified, of which 48 *NF1* mutations (missense, nonsense, and frameshift) were predicted to have a damaging effect [20]. Forty-five patients had independent *NF1* mutations [20]. In a further 15 (25%) cases, the *NF1* mutation co-occurred with mutations in other oncogenes: *BRAF* (p.C685S, p.G469V), *ERBB2* (p.S310Y, p.V308L), *KRAS* (p.G12D, p.G12V, p.G13C, p.G13D), *HRAS* (p.Q61H), and *NRAS* (p.G13R).

*TP53* (tumor protein P53) and *STK11*/*LKB1* (serine/threonine kinase 11) were the most common mutated suppressor genes found in tumors with a mutation in *NF1* or *KRAS* genes. Furthermore, *TP53* mutations or double-allelic deletions in this gene were significantly more frequent in patients with the *NF1* mutation (33/51, 65%) than in the *KRAS* mutation cohort (46/132, 35%) [19]. In our patient, in addition to the *BRAF* and *NF1* mutations with a known pathogenicity status, *TP53* (p.H179R), *FANCC* (p.Q13*), *NOTCH1* (p.Y708fs*9), *EPHB1* (p.R56L), and *TET2* (p.N752fs*59) variants were observed. Moreover, a variant with an unknown significance of pathogenicity was found in the *CDKN2A/B p16INK4a* (*cyclin-dependent kinase inhibitor 2A/B*) gene (p.D108G). These genes are mainly described as tumor suppressors, although this is debatable in some cases. Activating *NOTCH1* mutations may be present in 10% of NSCLC patients, while a decreased expression of NUMB (a negative regulator of NOTCH) is seen in 30% of NSCLC patients [20]. It is suggested that NOTCH1 expression varies according to the histopathological subtype, and the overexpression of NOTCH1 is associated with tumor progression and poor prognosis in NSCLC. However, other studies have shown that NOTCH1 overexpression is related to the inhibition of lymph node metastases and the progression of non-small-cell lung cancer [21,22]. Mutations in the *CDKN2A/B p16INK4a* tumor suppressor gene are some of the best-described mutations and have been implicated in many tumor types [23]. The identified p.D108G variant in this gene in the COSMIC database has a pathogenic (somatic lesion) status. However, there are no reports on the consequences of this mutation on protein functionality. Changes in codon D108 (D108H, D108Y) induce the destabilization of the cyclin-dependent kinase inhibitor and the loss of cell cycle control [24].

Free-circulating tumor DNA from specimens collected from our patient underwent sequencing using FOUNDATIONONE^®^ LIQUID CDx. The assay confirmed the presence of the previously mentioned mutations. Moreover, another alteration in the *TP53* gene was identified: c.733G>A (p.Gly245Ser). In hereditary cancer syndromes and Li-Fraumeni syndrome, it is described as a germinal lesion with a pathogenic status [25,26]. However, in squamous-cell lung carcinoma, is described as likely pathogenic and has a somatic lesion status [27]. The variant c.536A>G (p.His179Arg) in the *TP53* gene, identified in both tumor and liquid biopsy material, has a status of conflicting interpretations of pathogenicity in the ClinVar database. Numerous reports indicate that this mutation is pathogenic and this somatic variant occurs in numerous cancers, including small-cell lung cancer and squamous-cell lung cancer [27].

There are indications that co-occurring changes can be more precise biomarkers of therapeutic response than monogenic predictors [27]. The molecular changes in NSCLC patients with *BRAF* mutations include mutations in the following genes: *TP53* (53.3%), *STK11* (16.2%), *ATM* (*ataxia*-*telangiectasia mutated* serine/threonine kinase) (5.8%), *NF1* (6.9%), *PIK3CA* (*phosphatidylinositol-4,5-bisphosphate 3-kinase catalytic subunit alpha*) (6.6%), *KEAP1 (Kelch-like ECH-associated protein 1*) (6.6%), *MYC* (*MYC proto-oncogene, BHLH transcription factor*) (10.8%), *NKX2-1* (*NK2 Homeobox 1*) (7.3%) [28]. These data concern adenocarcinoma, while our patient was diagnosed with suspected lung squamous-cell carcinoma, and *BRAF* mutations in this histological type are extremely rare (0.3%). Mutations in the *BRAF* gene appear slightly more frequently in lung squamous carcinoma than in other squamous neoplasms [29,30,31,32]. The histopathological examination of the lung tumor in our patient did not exclude that the point of origin of the tumor may be outside the lungs. This may be indicated by the ambiguous immunohistochemical examination of the poorly differentiated squamous-cell metastatic tumor in the kidney (p40 (+), p63 (+), CK7 (+), CK5/6 (+), Vimentin (+), CD10 (+/−), GATA3 (−), Napisn A (−), CK20 (−), PAX8 (−), TTF1 (−), Ki67/MIB1 (+++ in 80% of cells)). Difficulties in immunohistochemical diagnosis, often not giving a clear answer as to the tissue of origin of the metastasis, are often observed in Cancer of Unknown Primary (CUP) [33]. Bochtler et al. [34] have studied 252 CUP [34]. Fifty-four patients from the earliest cohort were analyzed retrospectively, and the NGS results were available immediately after diagnosis in 198 patients. They identified mutations in the following genes: *TP53* (49.6%), *CDKN2A* (19.0%), *NOTCH1* (14.1%), *KRAS* (23.4%), *FGFR4* (14.9%), and *PIK3CA* (10.7%). In a sub-cohort of 198 patients, *BRAF* mutations were present in 3.5% of the population [34].

In our patient, we also found a pathogenic germline variant in the *FANCC* (*FA Complementation Group C*) gene at codon 13 of the frameshift with the termination type (c.37C>T, p.Gln13Ter). Mutations in the *FANCC* gene are associated with Fanconi anemia, a disease with chromosomal instability, hypersensitivity to DNA cross-linkers, a huge number of chromosomal breaks, and defective DNA repair. Moreover, the presence of mutations in the *FANCC* gene increases the predisposition to inherited cancers and increases the risk of the development of pancreatic, colorectal, breast, and lung cancers. Rao et al. described a case of a 55-year-old patient diagnosed with adenocarcinoma of the lung treated with surgical resection and chemotherapy and sequentially appearing colorectal adenocarcinoma. After polypectomy, squamous-cell renal carcinoma was diagnosed. The researchers intended to discover the primary tissue for the neoplastic lesions. After an immunohistochemical examination of these three tumors, it turned out that the results were inconclusive, as in our case described here. The researchers performed sequencing covering 520 cancer-related genes in three cancer samples. They found that the mutational profiles of the lung and kidney cancer tissues were very similar and different from the colorectal cancer tissue. Based on the sequencing data, the researchers concluded that the lung and colon cancer were primary tumors, while the kidney tumor was a metastasis of lung cancer [35]. A mutation in *FANCC* (*FANCC* p.W113X) was observed in all three cancer samples, confirmed as a germline change identified in the patient’s lymphocytes. The same *FANCC* variant was identified in the patient’s son, who was healthy at the time of the tests [35]. What is more, the patient’s father had colorectal cancer, which led to the diagnosis of hereditary cancer syndrome.

*FANCC* is involved in mechanisms that maintain genome stability and is considered as a tumor suppressor. The inactivating mutation p.Gln13Ter identified in our patients is a trigger of the elimination of suppressor activity, which may be associated with a worse clinical course of the disease. Moreover, patients with *FANCC* gene mutations have a well-documented increased risk to develop acute myeloid leukemia (AML) and myelodysplastic syndrome (MDS) [36,37]. It can be assumed that the development of MDS in our patient was caused by a hereditary mutation in the *FANCC* gene. The first symptoms of MDS also occurred in the patient’s daughter, who has not been genetically tested so far.

## 4. Conclusions

It appears that, in the clinical case described here, the inactivation of tumor suppressors facilitated tumor development and may have been involved in co-morbidities (MDS). Although these changes did not clearly qualify for molecularly targeted therapies, they could indicate a poorer prognosis as well as a shortened time to disease progression and overall survival [10,38]. The mutations in two genes—the *BRAF* oncogene and the *NF1* tumor suppressor gene—were the reason for the use of dabrafenib and trametinib treatment. The patients achieved short-term disease stabilization. This proved that coexisting mutations in these genes affect the disease course and treatment efficacy. Attention had to be paid to the patient’s comorbidities, including MDS, which complicated treatment. The mutation identified in the *FANCC* gene may suggest a genetic predisposition to cancers and MDS development in our patient. It is known that patients with Fanconi anemia are at a very high risk of bone marrow failure, MDS, leukemia, head and neck squamous-cell carcinoma, and other solid tumors [39]. The patient experienced deterioration related to the MDS course and died despite the dabrafenib and trametinib treatment with an apparent decrease in tumor size.

## Data Availability

The data presented in this study are available on request from the corresponding author.
